# Heterogeneity of Airborne Virus Transmission in the Built Environment: A Narrative Review

**DOI:** 10.20411/pai.v11i2.1049

**Published:** 2026-08-19

**Authors:** Yizhi Zhang, Kathryn C. Krupinsky, Adam S. Lauring, Linsey C. Marr

**Affiliations:** 1 Department of Civil and Environmental Engineering, Virginia Tech, Blacksburg, Virginia; 2 Department of Microbiology and Immunology, University of Michigan, Ann Arbor, Michigan; 3 Department of Internal Medicine, University of Michigan, Ann Arbor, Michigan

**Keywords:** SARS-CoV-2, Respiratory Particles, Superemitter, Ventilation

## Abstract

The COVID-19 pandemic accelerated recognition of airborne transmission of respiratory infections. Despite improved mechanistic understanding, our ability to predict the risk of infection in a given scenario remains limited, owing, in part, to high heterogeneity in factors involved in transmission. The goal of this work is to quantify variability in factors that control risk of airborne virus transmission in the built environment. Using SARS-CoV-2 as a model, we conducted a narrative review on transmission via inhalable respiratory particles (≤100 μm). We divided transmission into 3 key processes: emissions of virus in respiratory particles from an infected individual (the source) into the air; transport and decay of virus in the environment; and deposition and infection in a new host (the receiver). Among the source-related factors, we found large variabilities spanning 7 or more orders of magnitude. For example, the rate of respiratory emissions from an infected individual ranges from ~1 to > 10^7^ particles per second, depending in part on the type of respiratory activity and individual physiological factors. Unexplained inter-individual variations, such as those defining “superemitters,” introduce additional uncertainties. We also found considerable variability in the physical and biological decay of virus-laden particles as they move from the source to the receiver. The air exchange rate, which controls physical loss of particles by ventilation, ranges from ~0.01 to > 20 air changes per hour in typical buildings. Lastly, upon inhalation, the efficiency and site of virus deposition vary with the physiological state of the receiver, whose immunological status further influences whether infection is established. Overall, we found the largest variability in the rate of respiratory particle production and viral load of respiratory fluid. These 2 factors determine the amount of virus released into the air by an infected person, which is a critical indicator of the potential for onward transmission. Predicting the risk of infection in a specific scenario remains challenging due to the combined effects of variability in key determinants.

## INTRODUCTION

As the COVID-19 pandemic unfolded, the spread of SARS-CoV-2 challenged traditional notions of “droplet” and “airborne” transmission. Physicians, epidemiologists, virologists, and aerosol scientists came together to clarify routes of virus transmission. Airborne transmission, now defined as occurring through inhalation of pathogens in the air, can occur at both short range and long range from an infected individual and involves particles much larger than previously thought [1, 2]. Despite our improved, mechanistic understanding of airborne transmission, we are still unable to predict with certainty whether transmission will occur in a certain scenario. Why might hours spent in a small room with an infected individual yield no transmission, whereas a game of poker at a casino leads to several new cases, or vice versa?

The contagiousness of a disease is often described in terms of the basic reproductive number, R_0_, which represents the average number of secondary cases caused by an infected individual in an immunologically susceptible population. The pooled R_0_ for SARS-CoV-2 is estimated to be 3.32 [3]. The distribution of individual reproductive numbers is often right-skewed, or over-dispersed [4]. The dispersion parameter *k*, reflecting the level of variance around R_0,_ describes the level of heterogeneity in transmission. Specifically, if every primary case (ie, the originally infected individual) transmitted the disease to an equal number (R_0_) of people, then *k* approaches infinity, and transmission is homogeneous [5]. In contrast, if each primary case transmitted the disease to a wildly varying number of others, then *k* is less than 1. Transmission of SARS-CoV-2 is highly heterogeneous. The estimated mean values for *k* range from 0.04 to 2.97, with 93% of the studies reporting mean values lower than 1 [5]. While *k* may be interpreted to pertain only to variability in the primary case, transmission also depends on environmental parameters and susceptibility of the secondary cases.

The occurrence of superspreading events is consistent with a low value of *k* and supports the inherent heterogeneity in the risk of airborne disease transmission. During the well-documented Skagit Valley Chorale superspreading event in March 2020, 53 out of 61 attendees became infected [6]. Outbreaks at gyms in Hong Kong and Hawaii led to an estimated 101 and 21 cases, respectively [7, 8]. On the other hand, an infected instructor at a gym in Virginia led to no known secondary cases [9], demonstrating that gyms are not necessarily high-risk settings. Within households, the secondary attack rate was estimated at 19% in 2021, but surely most people know of anecdotes where a sick person transmitted the infection to anywhere from 0 to all co-inhabitants [10].

**Figure 1.**
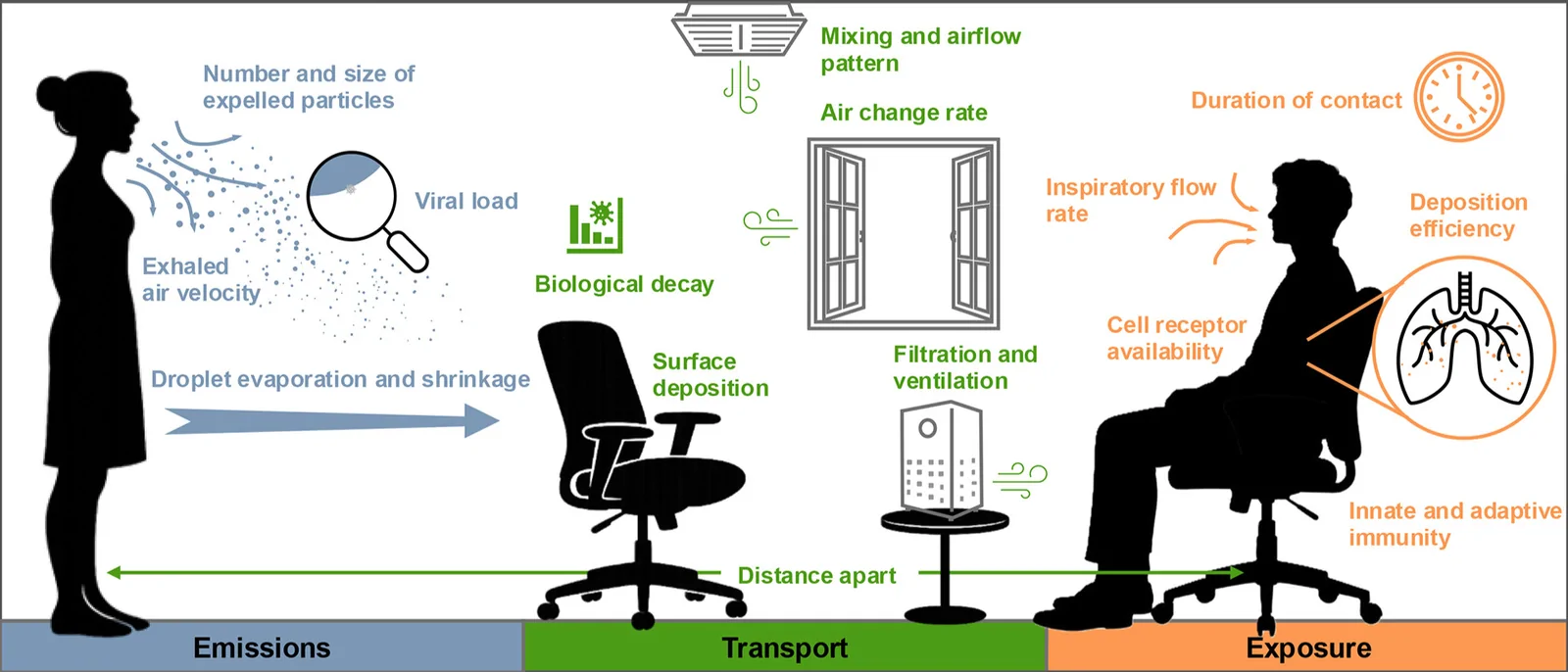
Factors contributing to heterogeneity in the risk of airborne virus transmission in a room, from an infected individual (source on the left) to a new host (receiver on the right). The factors are separated into 3 main processes: emissions, transport, and exposure.

The aim of this work is to identify and characterize variability in host and environmental factors that contribute to the risk of airborne virus transmission and lead to high heterogeneity in this risk. Using SARS-CoV-2 as a model, we focus on transmission by inhalable respiratory particles (≤100 μm) within a room, from one infected individual whom we call the “source” to a new host whom we call the “receiver” (Figure 1). The risk of transmission depends on numerous factors that we address in an order that physically tracks the virus from source to receiver: emissions into the air from the infected individual, transport through the air, and exposure, which for the purposes of this analysis, encompasses inhalation followed by deposition in the respiratory tract and establishment of infection. Transmission through large, ballistic droplets (particle diameter > 100 µm) and fomites is not addressed here.

## EMISSIONS

The first stage in airborne transmission is the generation and expulsion of respiratory particles from an infected individual. Virions are not ejected naked into the air; rather, they are carried in particles of respiratory fluid that are generated during respiratory activities, such as talking, singing, and coughing. The physical and biological properties of these expelled particles affect the likelihood of transmission. The jet or cloud of air carrying the particles also affects transmission and is addressed in the subsequent section on transport.

### Physical Properties of Respiratory Particles

Respiratory particles in the inhalable size range can be subdivided into a coarse fraction (5–100 μm), which remains suspended in the air for 5 seconds to ~1 hour before falling to the ground from a height of 1.5 meters, and a fine fraction (< 5 μm), which remains in the air for over 1 hour before falling to the ground from this height [1]. Their fate, including distance traveled, rate of removal, and likelihood of deposition if inhaled by another person, is related to size. Thus, both the size and number of exhaled respiratory particles influence the risk of transmission.

The physical properties of respiratory particles are affected by generation mechanism, site of origin, exhalation flow rate, and intensity of vocal tract movement. During respiratory activities, particles are generated from the surface rupture and aerosolization of mucosal fluids (ie, saliva and respiratory tract lining fluid) [11, 12]. Such rupture occurs as a result of airway expansion and deformation, respiratory airflow, and the movement of laryngeal and oral structures [12–15]. Breathing alone generates fewer, smaller particles compared to other respiratory activities. For vocalizing activities, additional factors like pitch, vocal intensity, and phonetic characteristics of the language spoken compound the variability [12, 16–18]. For example, loudness is positively associated with the number of particles generated. Compared to quiet speech, talking at an intermediate or loud voice generates on average 2–13 times more particles (> 0.5 μm), and the same trend is observed for loud vs normal singing [16, 17, 19]. Interestingly, the particle size distribution exhibits little variability across different vocalization activities. Specifically, talking and singing produce particles of similar size distribution regardless of loudness level [13, 17].

Fluid properties, such as viscosity and surface tension, influence particle production. These properties depend on the chemical composition of the fluid, which varies with the site of origin (eg, saliva in the oral cavity vs airway surface liquid along the respiratory tract) and state of disease [11, 20, 21]. The viscosity of respiratory fluid is highly variable; it can range over 4 orders of magnitude from 1 to 7 × 10^4^ mPa·s [11, 22]. By manipulating surface tension of the fluid, researchers demonstrated that nebulized saline reduced respiratory particle emissions by ~70% in high-producer participants, whereas surfactant increased emissions [23].

**Figure 2.**
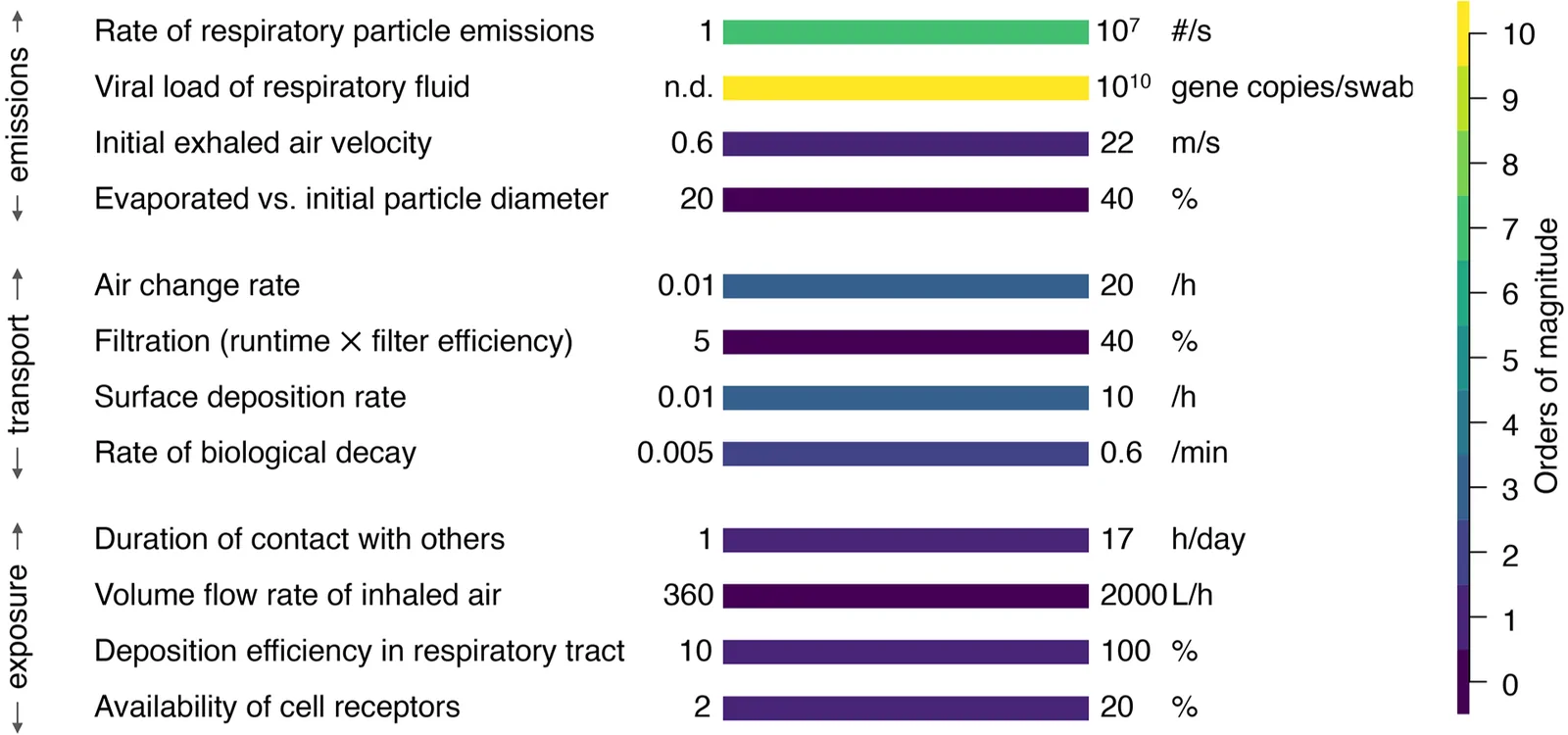
Variability in key factors affecting the risk of transmission, grouped in the order that physically tracks the virus from source to receiver.

Related to the physical factors that directly affect respiratory particle generation, physiological variables including age, sex, and health status can have indirect effects on particle generation. For example, children typically generate fewer particles because of their smaller laryngeal structure, fewer alveoli, and lower pressure in the subglottal system [24]. Some studies have reported an association between age, body mass index, and respiratory particle generation, but these findings are not consistent across the literature [13, 17, 25]. For particles emitted during breathing, inter-individual variability spans 3 orders of magnitude even within populations that share similar physiological characteristics [13, 19, 25]. Some individuals, “speech superemitters,” consistently emit more (eg, one order of magnitude higher than sample average) particles during vocalizing activities [13, 17, 19]. High-momentum events like coughing and sneezing are often associated with greater variability. While not directly transferable to SARS-CoV-2, particle (0.3–10 μm) counts from influenza patients demonstrated substantial variability (400–516,800 particles per cough), with a narrower range observed after recovery (300–362,700 per cough) [26]. However, researchers have not yet determined factors that would enable prediction of who might be a superemitter. Summarizing across these studies and assuming a breathing rate of 6 L per minute [27] to adjust between different ways of reporting results, we conclude that the emission rate of respiratory particles can span 7 orders of magnitude from 1 to > 10^7^ particles per second (Figure 2).

Upon release from the nose and mouth, particles begin to shrink due to evaporation of water, which is driven by the transition from the very humid respiratory tract to drier indoor air. The extent of size change depends on the particle’s initial size and the humidity of the surrounding air. For fine particles, this process happens within seconds, whereas for larger ones, it takes longer [28]. If the particles have a chance to reach equilibrium before they fall to the ground, their new diameter will be ~20% to 40% of the original size under most indoor conditions [29].

### Biological Properties of Respiratory Particles

A critical biological property of respiratory particles is their viral load, or the number of virions they carry. Genomic viral load is assessed by quantitative polymerase chain reaction (qPCR) in terms of gene copies per volume of fluid, whereas infectious virus is quantified by culture-based techniques like plaque assay. The amount of infectious virus in a sample is usually far less than the number of gene copies detected, and the probability of culturing live virus from PCR-positive samples (Ct < 35) is estimated to be lower than 10% [30]. A common sample type is a nasopharynx (NP) swab, often considered the gold standard for clinical diagnosis of infection. The amount of respiratory fluid collected by the swab is highly variable. After the sample is collected, the swab is placed in viral transport medium, and viral load is reported in terms of the volume of the fluid, which contains a small amount of respiratory fluid diluted in a much larger volume of medium. Therefore, this measure of genomic viral load concentration is lower than the actual viral load concentration in respiratory fluid.

One of the challenges in predicting virus transmission is the uncertainty stemming from temporal and individual-level variabilities in viral load [31]. For SARS-CoV-2, genomic viral load usually peaks at 0–5 days post onset of symptoms (dpos) and remains detectable for roughly 2 weeks afterward [32–34]. Viral RNA has also been found among samples from asymptomatic and pre-symptomatic individuals, who contribute substantially to transmission [32, 35–37]. In a series of swab samples taken from 9 patients, the average genomic viral load decreased from 6.76 × 10^5^ RNA gene copies per swab during 0–5 dpos to 3.44 × 10^5^ afterward [33]. Overall, studies reported NP swab results spanning up to 10 orders of magnitude (not detected to 10 × 10^10^ gene copies per swab) across different disease stages [33, 34, 38–40].

Detection of infectious virus in air samples presents significant challenges owing to low concentrations and difficulty in preserving virus viability [41]. Only a small subset of studies have attempted to culture SARS-CoV-2 in exhaled breath [42–45]. In one such study, exhaled breath was collected into liquid medium, and viable virus was detected at concentrations of 7.9 × 10^2^–2.5 × 10^4^ TCID_50_/mL from 10-minute talking and singing events [42]. However, samples from most (13 out of 16) of the COVID-positive participants were culture-negative [42]. Other similar studies reported even lower (< 5%) positivity from their patients [43, 45]. Notably, many additional studies were not able to culture SARS-CoV-2 from exhaled breath samples, despite confirmation of infection based on detection of viral RNA in the samples, or of infectious virus in NP swabs [38, 46, 47].

### Size-resolved Viral Load of Respiratory Particles

Perhaps more relevant to transmission than viral load in NP swabs is the amount of virus carried by exhaled respiratory particles [48]. Knowing how virions are distributed across particles of different size would improve our ability to predict transmission. For example, if nearly all virions were present in larger particles, then we would expect transmission to occur mainly within a limited distance of the source. A handful of investigations have simultaneously measured both the size distribution and viral load of respiratory particles.

Despite differences in sampling approaches, there is a general agreement that viral content is not proportional to particle volume. In exhaled breath samples from 13 patients during 15-minute talking events, the average amount of SARS-CoV-2 RNA gene copies was 417 (IQR 191.2–979.5) in fine particles (≤ 5 μm), and far fewer (0 copies, IQR 0–77.8) in coarse particles (> 5 μm) [46]. Measured with a different sampling device, gene copies in particles emitted by a single patient peaked within the size range of 0.94–2.8 μm [49]. In both studies, 90% of total RNA gene copies were detected from particles smaller than 4.5 μm [46, 49]. However, the paucity of such studies makes it difficult to draw further conclusions regarding size-dependent virion distribution patterns.

## TRANSPORT

The second stage of airborne transmission is the movement of particles from the source to the receiver. During one breathing cycle, the expelled particles are first carried in a jet of air expelled from the nose and/or mouth and then dispersed in a puff-like fashion, resulting in a trumpet-shape dispersion pattern [50, 51]. Short-range airborne transmission occurs when the receiver inhales particles directly from the exhaled air stream [52]. Long-range airborne transmission may occur when the receiver is outside the trumpet and inhales infectious particles that are carried by air currents throughout the room.

Because short-range and long-range transmission are driven by different factors, we treat them separately below. Although this threshold between short- vs long-range transmission is neither sharp nor fixed, it is convenient for thinking about transmission at different distances. This threshold distance is a function of many variables, including exhaled air velocity, position and orientation of the source, and pattern of the surrounding airflow. While a 1.5–2 m cutoff is commonly used in modelling studies for talking events, the distance for coughing and sneezing could be as large as 2.5–3 m [52–55].

### Short-range Transport

Short-range transmission occurs within seconds after respiratory particles are emitted by the source [52]. At this initial stage, particle dispersion is driven mostly by the expiration jet and turbulent mixing [53]. Thus, distance and direction of both the source and receiver can introduce uncertainties in the risk of short-range transmission. While large droplets follow ballistic trajectories and exit the exhalation jet quickly, smaller particles are affected by the initial flow rate and velocity of the exhaled air, by the surrounding air conditions, and by body thermal plumes [52, 53, 56]. Both the flow rate and air velocity are highly variable, owing to differences in activity level, type of respiratory event, and physiological factors like the size of mouth opening. Initially, exhaled breath can travel at a volume flow rate ranging from 0.25 to 8.5 L per second and a velocity of 0.6–22 m per second [27, 57, 58].

### Long-range Transport: Physical Movement

Long-range transport of respiratory particles is dictated by indoor air movement, which is driven by pressure or thermal gradients from various sources including human thermal plumes, and air movement through windows, doors, and heating, ventilation, and air conditioning (HVAC) systems. The velocity of indoor air typically ranges from 5 to 20 cm per second [59, 60]. Thus, during the ~30 minutes that a 5 μm particle is suspended in air before it settles to the ground, it could travel a horizontal distance of 100–400 m. Governed by indoor air currents, this travel trajectory is often a non-linear, meandering path rather than a straight line. Depending on particle size, long-range transport can occur over minutes or even hours. During this time, exhaled particles and any viruses they contain are subject to a range of physical removal processes, mainly deposition to surfaces, ventilation, and filtration.

Particles can be removed from the air by deposition onto indoor surfaces. The rate at which this occurs is characterized by the deposition velocity, which has units of distance per time, and the deposition rate, which has units of particle number per time. The deposition rate depends on particle size and is affected mainly by indoor airflow conditions and surface roughness [61]. Consistent with theoretical expectations, a study in 6 Hong Kong homes found that particles in the size range of 0.54–0.78 μm had the lowest deposition velocity (0.311 × 10^-4^ m per second), while particles smaller or larger than this range had higher deposition velocities (0.6 ×10^-4^ –1.16 ×10^-4^ m per second) [62]. Other field studies have reported considerable variability in the deposition rate. For example, the deposition rate spans two orders of magnitude (0.01–1 per hour) for particles of a 0.2 μm diameter [63]. Overall, the deposition rate varies from 0.01 to 10 per hour for particles < 10 μm across different airflow and furnishing conditions [61, 63]. This means that removal of viruses from the air by deposition can vary by 3 orders of magnitude depending on the carrier particle size and local environmental conditions.

Virus-laden particles can also be removed from a room by ventilation, which is quantified by the air change rate in terms of air changes per hour (ACH). ACH describes how quickly outdoor air is supplied to a room. In hospitals, the ventilation rate is typically 6–12 ACH, meaning that “new” air is supplied to the room every 5–10 minutes. Ventilation may occur through natural means such as opening doors and windows, or through mechanical HVAC systems. The median air change rates in a sample of residential and office buildings across 3 cities in the United States was found to be 0.7 ACH and 1 ACH, respectively [64, 65]. Studies have found that mechanically ventilated classrooms typically have higher air change rates (1–> 8 ACH) than naturally ventilated ones [66–68]. These rates vary daily and seasonally, and opening windows and using fans can increase the air change rate by a factor of 2–70 [68–70]. In the absence of natural or mechanical ventilation, infiltration of air through leaks in the building envelope becomes more important. Newer buildings with tighter envelopes typically exhibit lower infiltration rates, while the air change rate in a given space can vary substantially as outdoor conditions change [71–73]. Overall, the air change rate in typical residences, office spaces, and classrooms can range from 0.01 to over 20 ACH [68, 69, 72, 74]. Thus, the rate of virus removal by air movement from indoors to outdoors varies by at least 3 orders of magnitude among rooms and can fluctuate temporally with outdoor conditions.

Pathogens can also be removed from the air by filtration through a central HVAC system or a standalone portable air cleaner (PAC), often referred to as an air purifier. The effectiveness of air filtration is highly variable, as it depends not only on the device, but also on its operating patterns and surrounding environmental conditions [75, 76]. An HVAC system that runs only when needed to satisfy heating or cooling demand would only be filtering air for 20% to 27% of the time during periods of mild outdoor temperatures [77]. Common high-efficiency filters (ie, minimal efficiency reporting value [MERV] 8 and above) have initial efficiencies ranging from < 10 to ~100%, depending on particle size and air velocity [78]. Other issues like bypass, energy consequences, and decrease in filter performance as a result of dust loading further introduce uncertainties in system performance [75]. PAC performance is typically measured in terms of the clean air delivery rate (CADR) that reflects the volume of filtered air delivered into the space [79]. While some studies measured CADRs that are comparable to the products’ claims (eg, [70, 79, 80]), others found lower CADRs in real-world use (eg, [81, 82]). Noise level, placement strategy, and filter degradation over time present constraints on optimal PAC performance [82–84]. Overall, a PAC can have CADR anywhere from ~30 to > 1,400 m^3^ per hour depending on design and operation [80, 84]. This means that in a typical classroom with a volume of 300 m^3^ (10 × 10 × 3 m) and a baseline air change rate of 3 ACH, using a PAC can provide an additional equivalent 0.1–4.7 ACH, enhancing virus removal from air by a factor of 1–2.

### Long-range Transport: Biological Decay

Even if a virus is still physically present in the air, it may experience biological decay, meaning that it has lost its ability to infect a host cell. Its envelope, glycoproteins, or genetic material may be damaged. The biological decay rate of a virus depends on environmental factors like temperature, relative humidity (RH), ultraviolet (UV) radiation, and the composition of the fluid in which the virus is embedded [85]. Biological decay is typically quantified in terms of a decay constant with units of inverse time, or the time to achieve 50% (half-life), 90%, or 99% reduction.

Indoor temperature typically falls within the range of 20–30 °C and is not believed to be a dominant factor in the variability of virus inactivation indoors. On the other hand, RH typically varies between 20% and 80% and has been consistently shown to be an important factor in virus inactivation. For SARS-CoV-2 and certain other enveloped viruses, infectivity often shows a U-shaped dependence on RH, with greater persistence at low (< 40%) and high (> 80%) RHs and more rapid decay at medium RH (40%–80%) [86]. Assuming first-order decay, viral inactivation can be characterized by a rate constant, *k*, with units of inverse of time. Studies have reported *k* values for aerosolized SARS-CoV-2 ranging from 0.005 to 0.6 per minute [87–89]. This means that the lifetime of the virus against inactivation can vary by three orders of magnitude, depending on the surrounding humidity.

Germicidal ultraviolet (gUV) irradiation at a wavelength of 254 nm is well-established as a method of inactivating airborne viruses, including SARS-CoV-2 [90–93]. Interest is growing in far-UV irradiation at a wavelength of 222 nm, which unlike traditional gUV, should be safe for skin and eyes [94, 95]. Building on earlier literature on other coronaviruses, the far-UV susceptibility of SARS-CoV-2 in air was recently demonstrated in laboratory studies [96, 97]. Overall, the measured far-UV inactivation rate constant for airborne coronaviruses ranges from 4 to 14.26 cm^2^/mJ [95, 97–100]. Although findings from these chamber studies cannot be directly extrapolated to infer far-UV performance against airborne SARS-CoV-2 in real-world settings, the disinfection potential of far-UV light has been demonstrated in room-scale experiments using surrogates [96, 101–103].

### Long-range Transport: Position Between the Source and the Receiver

For the sake of mathematical simplicity, air is often assumed to be “well-mixed” in indoor air quality studies. This assumption is also required of the fundamental mass-balance equation that has been applied in many field and modeling studies. However, recent evidence shows that this assumption is often not true, and the spatial distribution of indoor air pollutants is not homogenous across the space of interest [104]. Similarly, the distribution of expelled respiratory particles is likely to vary spatially in a room and is dependent on the direction and magnitude of the air trajectory between the source and receiver. Within a given space, air movement patterns are influenced by outdoor conditions, window/door-related parameters, indoor surface temperatures, and operation of HVAC system and other air-cleaning devices, and the patterns are often dynamic. Air flow patterns that carried airborne virus from the source toward receivers have been implicated in observational studies of SARS-CoV-2 transmission indoors [105, 106].

The particle dispersion, or distribution, pattern in a room has been investigated in both field and modeling studies. In field studies, a tracer is typically released into a room to artificially elevate pollutant concentrations, and measurements are collected at various locations across the room, while modeling studies often rely on computational fluid dynamics (CFD) simulations. Dispersion patterns can then be analyzed by tracking tracer movement through the space, and the degree of mixing is assessed from the spatial variability of measurements distributed across the space.

Researchers have found varying mixing and dispersion patterns even in the same space. First, ventilation has a fundamental role in shaping the dispersion and transport of pollutants in a room [83]. Mixing patterns in naturally ventilated classrooms are highly variable as a result of different door and window operating conditions [69]. To a lesser extent, the operation mode of the central HVAC system affects the degree of mixing in mechanically ventilated rooms [107]. An overhead system operating in heating mode (as opposed to cooling or fan-only mode) is more likely to result in poor mixing due to thermal stratification [108, 109]. As a result of imperfect mixing of indoor air, varying levels of exposure are expected, depending on the position of the source and the receiver. This is supported by the presence of “hotspots” where pollutant concentrations may be up to 10 times higher than at other locations in the same space [107, 108, 110]. Furthermore, locations of maximum and minimum exposure may vary with air change rate [110]. In summary, in a room that is assumed to be well-mixed, the receiver’s exposure to airborne virus may vary by an order of magnitude, depending on the person’s location within the room relative to vents, doors, windows, and furniture and the ventilation conditions. A limitation of such analysis is that many of the studies used tracer gas or salt particles as proxy for airborne viruses. Thus, predicted concentrations might not directly translate to the quantity of infectious virus.

## EXPOSURE

The final step in our treatment of transmission is exposure, defined here to encompass inhalation and deposition of the virion in the respiratory tract, followed by initiation of infection. After being emitted into the air and transported to the receiver, the virus may be inhaled. The chances of this occurring, of the virus depositing along the respiratory tract, and of the deposited virus initiating infection are a function of the receiver’s behavior and physical and biological characteristics. In other words, when 2 people are exposed to the same air, particle deposition patterns and the initial immune response are likely to differ between the individuals, producing differing risks of infection.

### Behavior

Naturally, a longer duration of exposure produces greater risk of transmission due to the larger number of virus particles inhaled. A study in Germany estimated that people can spend up to 17 hours per day in contact with others, with the majority of these contacts occurring indoors [111]. Sociological and epidemiological studies have estimated the time individuals spend together in different settings. For example, students were found to spend anywhere from 3 to 6 hours in the same classroom during a typical school day [112]. Staff-patient contact time was found to be highly variable (0.03 to ~1.5 hours per day) in a 4-day-long hospital study [113]. Furthermore, 16% of the staff members accounted for over 40% of both number and duration of contacts with patients and were categorized as “super-contacts” [113]. For short-range airborne transmission, variability in distance, head orientation, and body movement further complicates the risk of exposure [52].

### Deposition Pattern: Lung Morphology and Breathing Pattern

Inhaled particles can deposit onto the surface of the respiratory tract, or they may be exhaled without depositing. Particle deposition is size-dependent, with a minimum efficiency of ~10% for particles in the size range of 0.2–0.5 μm [114]. In contrast, particles that are much smaller or much larger have substantially higher deposition efficiencies, up to ~100% [114, 115]. Additionally, larger particles are more likely to deposit in the upper respiratory tract, whereas smaller particles have a greater chance of reaching and depositing in the lower respiratory tract [115]. Thus, the amount of virus that deposits in a particular region of the respiratory tract depends, in part, on the size of the particles carrying the virus, a property that is not known with great certainty.

Similar to how one’s respiratory particle emissions are in part a function of their age, sex, and other physiological parameters (eg, respiratory diseases that changes lung structure), the deposition pattern of particles along the respiratory tract also depends on these factors. Inhaled particle trajectories and the resulting deposition sites depend on individual lung morphology and breathing patterns, which may vary widely between individuals [114]. For example, children, whose lower respiratory systems are still developing, are expected to have different lung structure and deposition patterns from those of adults [116]. Breathing volume and frequency, which determine the total volume of air inhaled, can vary both between and within individuals. Nonetheless, adult tidal volume during normal breathing typically remains within the same order of magnitude. For example, measured tidal volumes ranged from 389±75 to 1463±210 mL in a study of 16 healthy participants [117]. Furthermore, both respiratory rate and volume are known to vary in response to neurophysiological and environmental stressors. Factors like exercise, heat, and stress can increase both the rate and depth of breathing [118]. In general, breathing frequency can increase up to 50% and tidal volume up to 200% in response to stressors, while the total volume of breathed air is estimated to remain within the same order of magnitude [118].

### Location of Infection and Non-specific Physical Barriers

SARS-CoV-2 initiates infection in 1 of 2 sites: the oropharynx or the nasal cavity. Both sites contain epithelial cells supportive of productive cellular infection; however, a virion must first reach these cells to establish infection, an action that can be inhibited by intrinsic defenses [119–121]. Each of these sites has key physical barriers that limit the number of virions that reach epithelial cells. One major barrier mechanism is mucociliary clearance, which traps foreign materials in mucus (produced by secretory cells) and eliminates them by ciliary-generated flow [122]. The effectiveness and rate of clearance can vary. Within the nasal cavity, mucus is rapidly replaced about every 10 minutes [123]. The rate within the oral cavity is not presently known. Aside from this mechanical role, mucins aid in biochemical clearance. The expression levels of these mucins can differ from person to person based on a variety of factors, leading to differing levels of clearance. For instance, prior work shows that mucin expression may be impacted by sex hormones (eg, estrogens), which can potentially explain some observed differences in infection establishment and dynamics between sexes [123]. In addition to mucociliary clearance, saliva also plays a role in limiting infection establishment [124]. Major salivary glands are found in the oropharynx; minor salivary glands are present in the nasal cavity. Differences in glandular types, location, and abundance can cause distinct shedding dynamics and, in turn, impact likelihood of infection establishment [119]. Finally, the microbiota of the oropharynx and the nasal cavity serves as an additional physical layer that interferes with the virion-epithelial cell interactions necessary for infection [125]. While less common, virions can directly establish initial infection in lower parts of the respiratory tract if they reach and deposit in this region [126, 127]. In these cases, establishment success depends on the precise landing location [126, 127]. While variability in barrier mechanisms is recognized, to our knowledge, it has not been quantified.

### Cell Tropism and Availability

If a virion surpasses non-specific physical barriers and reaches the underlying respiratory epithelium, it must find a cell permissive of infection. For SARS-CoV-2, this largely means reaching a cell expressing the canonical receptor, ACE2 [128]. Other receptors (such as KREMEN1 and ASGRI1) have also been shown to contribute to infection [129]. There may be numerous receptors with varying levels of permissiveness for infection, and the full receptor space is incompletely known [130, 131]. Effectiveness of infection following entry-related-interactions between host and viral proteins can further vary depending on host mutations (such as N720D in ACE2) [132]. SARS-CoV-2 permissive receptors are found on 3 cell types in the respiratory epithelium: goblet cells, ciliated cells, and basal cells [130, 133]. Relative levels of receptors vary based on cell type, age, and the genotype of individuals [122, 132, 134]. These receptor levels are further altered by location within the respiratory tract [135, 136]. The combination of these factors can explain observed differences in the tissue-specific replication rates and subsequent impacts on the probability of infection [122, 127]. The nasal cavity has an ACE2 positivity range of 2% to 20% [127]. Meanwhile, the percentage of ACE2 positive cells in the tracheobronchial compartment is much lower [137]. These differences are also dependent on cell type availability in each region of the respiratory tract [122, 127, 133, 137].

### Host Defense

Following initial cellular infection, SARS-CoV-2 must replicate and spread through a tissue to produce enough progeny to overcome stochastic extinction and establish host-wide infection. While beneficial for the pathogen, this replication and spread is not beneficial for the host. Thus, there are many host-mediated processes to prevent propagation of viruses. These processes can be tissue-specific (ie, autonomous from the rest of the body) or reliant on multiple organs/organ systems [131].

The host’s immune system has 2 arms: the innate system, which is immediate and non-specific, and the adaptive system, which is delayed in its action and pathogen-specific. Given the rapid nature of infection establishment, innate immunity is more relevant to transmission. One of the most fundamental host-mediated innate anti-viral mechanisms is upregulation of cell death pathways in infected and proximal cells [131]. This process is coordinated and accomplished in part through the release of cytokines produced in response to triggering of pathogen recognition receptors (PRRs) [138, 139]. Increased expression of PRRs can boost protection against infection and, therefore, early cytokine effector functions often coordinate upregulation of PRRs [138–140]. The effectiveness of this process can vary from individual to individual and lead to differences in the likelihood of infection establishment [141]. For SARS-CoV-2 infection, the most important cytokine is interferon (IFN) [121, 142, 143]. IFN is known to directly limit SARS-CoV-2 replication and, interestingly, its production can also upregulate ACE2, the canonical SARS-CoV-2 receptor [121, 143]. This cytokine is notably produced by epithelial cells in response to PRR triggering; however, dendritic cells (resident innate immune cells) are a major producer of IFN during early infection [134, 143]. Nonetheless, IFN alone does not fully explain observed establishment dynamics and thus is only part of relevant processes [121].

At any given time, resident alveolar and monocyte-derived macrophages are present in the respiratory tract [138]. The proportion of each varies from person to person and correlates with infection propagation and establishment potential [138]. Studies also show that early activation of basophils and eosinophils is important for protection against early infection [138]. Other innate cells augment intrinsic immune functions in a variety of ways. Natural killer (NK) cells produce cytokines and kill infected and proximal cells, with specific major histocompatibility complex (MHC) alleles changing SARS-CoV-2 binding specificity and subsequent activation potential [132, 138, 144, 145]. Neutrophils produce neutrophil extracellular traps (NETs), which directly inactivate virions and prevent infection propagation [146]. Innate lymphoid cells (ILCs) produce cytokines [147]. Hosts vary in their baseline availability of all of these cells and, subsequently, their ability to prevent infection spread [145, 147, 148]. One interesting additional cellular defense mechanism is emergency hematopoiesis. Emergency hematopoiesis is the response of hematopoietic stem cells to PRRs and pro-inflammatory cytokines leading to rapid differentiation into immune cell types that contribute to infection establishment. Rates of this process can vary widely, which leads to differences in the effectiveness of the response [149]. In SARS-CoV-2 naive individuals, adaptive immunity is not thought to play a large role in modulating infection establishment owing to its late timing as compared to infection establishment (1–2 days vs 7+ days) [150]. However, antibodies that are polymicrobial or anti-IFN may modulate the probability of infection establishment [119, 125, 132, 144, 151, 152]. Additionally, studies suggest that infection establishment may be altered by cross-reactive seasonal CoV antibodies [153]. Mucosal antibodies (from prior infection or exposure, or cross reactivity with other CoV) neutralize the virus and limit transmission; yet, their importance at time of infection is uncertain due to waning and variation in levels of cross reactivity [154–156]. SARS-CoV-2 vaccination shifts the importance of the adaptive system in infection establishment; however, specifics regarding its precise modulation are outside the scope of this review. Again, for host defenses, variability has been recognized, but the magnitude of variability is not well-known.

## CONCLUSION

The heterogeneity of SARS-CoV-2 transmission poses challenges for both individual-level risk assessment and population-level transmission predictions. Our review indicates that heterogeneity in airborne transmission is driven by variations in key parameters across the transmission pathway, from the expulsion of virus-laden particles into the air, to environmental transport and survival, and finally deposition and infection in a recipient. How does this variability translate to risk of infection? The Wells-Riley equation is frequently used to model the risk of infection, given inputs such as the amount of virus released into the air, the receiver’s minute ventilation, the ventilation rate of the room, and the duration of exposure [157]. As the equation is based on the Poisson distribution, the risk of infection increases monotonically with many of the factors described above but with diminishing marginal increases.

Among the quantifiable factors that affect the risk of transmission, variability commonly spans more than one order of magnitude. Notably, the rate of respiratory particle production and viral load of respiratory fluid span at least 7 and 10 orders of magnitude, respectively. Together, these two factors determine the amount of virus released into the air by an infected person, which is a critical indicator of the potential for onward transmission. Improving our ability to predict the risk of transmission in specific scenarios will require more precise estimates of how much virus an infected individual releases into the air, among other factors. Ultimately, understanding the mechanisms of transmission and the variability in factors that affect the risk of transmission will lead to improved ability to predict disease dynamics and selection of the most promising interventions.
